# Patients’ experiences and perceived outcomes of manual therapy: a qualitative systematic review with framework synthesis

**DOI:** 10.3389/fpain.2026.1767395

**Published:** 2026-09-11

**Authors:** Ying Zhang, Shasha Liang, Hong Chen, Ya'nan Sun, Changhe Yu

**Affiliations:** 1 Yun Gang Community Service Center, Beijing Genertec Aerospace Hospital, Beijing, China; 2 The First Clinical Department, Beijing University of Chinese Medicine, Beijing, China; 3 Tuina and Pain Management Department, Beijing University of Chinese Medicine Affiliated Dongzhimen Hospital, Beijing, China; 4 Xuanwu Hospital, Capital Medical University, Beijing, China

**Keywords:** bio-psycho-social model, dynamic adaptation mechanism, manual therapies, multidimensional assessment, tactile-Mediated

## Abstract

**Objective:**

To systematically synthesize qualitative evidence on patient-reported experiences of manual therapies (including massage therapy, osteopathic manipulation, and craniosacral therapy) across biological, psychological, and social dimensions. We aimed to identify patient-perceived outcomes, contextual factors influencing the therapeutic process, and hypothesized mechanisms underlying patient experiences, thereby constructing an integrated conceptual framework centered on patient narratives.

**Methods:**

A systematic search of Chinese and English databases was conducted from inception to March 2024 for qualitative studies exploring patient experiences of manual therapies. Methodological quality was assessed using the COREQ (Consolidated Criteria for Reporting Qualitative Research) checklist and the CASP (Critical Appraisal Skills Programme) Qualitative Checklist. Best-fit framework synthesis was employed to integrate patient-reported outcomes within the COMET (Core Outcome Measures in Effectiveness Trials) framework. Contextual factors influencing patient experiences were organized under the contextual factors framework proposed by Marco Testa for physical therapy. A diagnostic and therapeutic model of “assessment-intervention-feedback” was constructed based on the mechanisms of dynamic adaptation and multisensory integration.

**Results:**

Fifteen studies were included. Manual therapies were found to significantly improve clinical symptoms, physical function, affective function, and quality of life. Simultaneously, background factors—including therapist characteristics and patient characteristics—were revealed as multidimensional regulatory mechanisms of therapeutic efficacy. A diagnostic-treatment system was constructed using tactile input as the nexus, dynamic regulation as the foundation, and multisystem synergy as the distinctive feature. Its four technical dimensions synergistically functioned throughout the diagnostic-treatment cycle, achieving integrated regulation of physiological-psychological-social systems.

**Conclusion:**

Manual therapy achieves multidimensional synergistic therapeutic effects through a dynamic coupling mechanism mediated by touch, integrating mechanical, neurological, and social dimensions. Its diagnostic and therapeutic system provides an innovative paradigm for the management of chronic diseases and the promotion of psychosomatic health. Future research should focus on optimizing personalized interventions by integrating artificial intelligence and wearable technologies, thereby facilitating the transition from empirical medicine to a precision science paradigm.

## 1 Background

Manual therapy, a non-pharmacological technique, is widely regarded as beneficial and recommended for a variety of illnesses, including lower back pain, neck pain, tension headaches, osteoarthritis, and fibromyalgia (1–8). Clinical studies have demonstrated that manual therapy enhances quality of life, treatment satisfaction, and physical, psychological, and social results (9–11). Qualitative research of manual therapy revealed that following treatment, patients had feelings of relaxation, better sleep, less stress, anxiety, and exhaustion, as well as increased vitality and stronger relationships (12). Another research found that the success of manual therapy depends on a number of factors, including practitioner expertise, techniques employed, patient expectations, communication between the patient and the practitioner, and environment variables like lighting and temperature (13, 14). Qualitative research prioritizes patients' experiences, perspectives, expectations, and treatment contexts, thereby enabling an expanded understanding of the domains in which manual therapy may be experienced as beneficial and the processes through which patients perceive positive changes. There is still a dearth of systematic synthesis of qualitative findings, despite the fact that there are already many qualitative studies based on patient interest groups that have addressed the aforementioned topics from different levels and dimensions.

The objective of this qualitative evidence synthesis is to develop a conceptual understanding of patients' experiences with manual therapy by systematically reviewing and synthesizing qualitative studies that explore patients' perspectives on safety, perceived outcomes, and contextual factors during manual therapy encounters. Through framework synthesis, we aim to identify the outcome domains that patients themselves consider meaningful, examine how treatment context shapes these experiences, and generate hypotheses about potential mechanisms underlying patients' reported experiences. It is anticipated that these findings will inform the development of patient-reported outcome frameworks, refine manual therapy approaches to better align with patient values, and offer directions for future qualitative inquiry and clinical practice. For the purpose of this review, manual therapy is defined as skilled, hands-on therapeutic techniques delivered by trained clinicians (including but not limited to physical therapists, chiropractors, osteopaths, and traditional medicine practitioners) involving the application of manual force to joints, muscles, nerves, or soft tissues. These techniques encompass a spectrum of approaches, from gentle joint mobilization and soft tissue manipulation to high-velocity thrust techniques, with the common goal of improving movement, reducing pain, and enhancing functional capacity. This definition was chosen to capture the breadth of patient experiences across diverse manual therapy modalities and professional contexts.

## 2 Methods

### 2.1 Literature search

Systematic retrieval was conducted in both Chinese and English databases, including China National Knowledge Infrastructure (CNKI), VIP Chinese Journal Full-Text Database, Wanfang Data Knowledge Service Platform, China Biology Medicine (CBM) disc, PubMed, Web of Science, EMbase, and Cochrane Library, covering literature from the database establishment to March 31, 2024. Given the scope of this review and the stability of the patient experience literature in this field, we did not update the search beyond this date. However, we acknowledge that more recent studies may have emerged since then, which could be incorporated in future updates. The search strategy combined MeSH (Medical Subject Headings) terms with free-text words, and the specific search strategies are detailed in Supplementary Appendix 1. The reference lists of included studies and qualitative research integration-related literature were used as supplementary search sources.

### 2.2 Inclusion and exclusion criteria

**Inclusion Criteria:**

Participants: Adults (≥18 years) who have received manual therapy delivered by trained clinicians, including but not limited to physical therapists, chiropractors, osteopaths, and traditional medicine practitioners. Manual therapy was defined as skilled, hands-on therapeutic techniques involving the application of manual force to joints, muscles, nerves, or soft tissues, encompassing a spectrum of approaches from gentle joint mobilization to high-velocity thrust techniques.

Phenomenon of Interest: Patients' experiences, perspectives, perceptions, and expectations regarding manual therapy, including perceived changes, treatment processes, contextual factors, and mechanisms as understood by patients.

Setting: Patients receiving manual therapy in any healthcare or clinical context, including inpatient, outpatient, community-based, or private practice settings.

Study Type: Qualitative research designs (phenomenology, grounded theory, ethnography, descriptive qualitative research, thematic analysis) or qualitative components within mixed-methods research from which qualitative findings could be independently extracted. All forms of qualitative data were eligible, including in-depth interviews, focus groups, participant observation, and open-ended survey responses.

**Exclusion Criteria:**

Systematic reviews, scoping reviews, or other secondary synthesis literature; Studies with insufficient reporting of qualitative data (e.g., absence of participant quotations or clearly articulated thematic findings); Studies not related to manual therapy; Studies published solely as conference abstracts without full-text availability; Studies focusing exclusively on clinician perspectives without patient-reported data.

### 2.3 Literature screening and data extraction

#### 2.3.1 Screening process

Literature deduplication and screening were conducted using NoteExpress reference management software. The screening process followed a two-phase approach:

Phase 1 (Title and abstract screening): Two reviewers (ZY and LSS) independently screened titles and abstracts against the inclusion criteria. Studies clearly meeting exclusion criteria or not meeting inclusion criteria were excluded. Studies with insufficient information to determine eligibility were retained for full-text review.

Phase 2 (Full-text screening): The same two reviewers independently assessed full-text articles. Reasons for exclusion at this stage were documented. Disagreements were resolved through discussion; when consensus could not be reached, a senior reviewer (YCH) made the final decision.

#### 2.3.2 Data extraction

Following the determination of included studies, two researchers (ZY and LSS) independently performed data extraction using a predefined, pilot-tested extraction form. Disagreements were resolved through discussion with the senior reviewer (YCH).

The data extraction form included:1)Basic study information: Title, authors, year of publication;2)Methodological characteristics: Qualitative approach/design, participant characteristics, data collection methods (interview, focus group, observation), analytical approach;3)Patient-reported findings: Themes, categories, or descriptive categories reported by the original authors regarding patient experiences, perceived changes, and contextual factors;4)Illustrative data: Key patient quotations that grounded the reported themes.

#### 2.3.2 Handling of Chinese and English language studies

Studies published in Chinese were analyzed in their original language by reviewers fluent in Chinese (ZY, LSS). Studies published in English were analyzed in English. During the synthesis stage, findings from Chinese-language studies were translated into English for framework mapping and thematic integration. Translation aimed to preserve conceptual meaning rather than literal equivalence. Key patient quotations from Chinese studies were translated into English and back-translated by an independent bilingual researcher to ensure accuracy. The cultural context of each study (e.g., differences in healthcare systems, manual therapy traditions, patient-clinician relationship norms) was explicitly considered during interpretation. Studies from different cultural contexts were not treated as directly comparable; rather, cultural variation was explored as a potential source of divergent findings.

### 2.4 Quality assessment

#### 2.4.1 Reporting quality assessment

The reporting transparency of included studies was assessed using the 32-item Consolidated Criteria for Reporting Qualitative Research (COREQ) checklist (15). COREQ was originally developed as a reporting guideline for primary qualitative research involving in-depth interviews and focus groups. We used it to evaluate whether studies adequately reported key methodological details—including researcher characteristics, participant selection processes, data collection methods, and analytical procedures—necessary for readers to assess the credibility and transferability of findings.

We explicitly acknowledge that COREQ is not a critical appraisal tool and does not evaluate methodological rigor, trustworthiness, or study quality *per se*. Its purpose in this review was to describe the reporting transparency of the evidence base, not to judge the inherent value of individual studies.

Two reviewers (ZY and LSS) independently assessed each included study against all 32 COREQ items, recording “Yes” (reported), “No” (not reported), or “Unclear” (insufficient information to determine). Discrepancies were resolved through discussion or consultation with a third reviewer (YCH). No study was excluded based on reporting quality alone.

#### 2.4.2 Methodological critical appraisal

To evaluate the methodological credibility of included studies, we supplemented COREQ with the 10-item Critical Appraisal Skills Programme (CASP) Qualitative Checklist (16). The CASP tool assesses whether: (a) the research design was appropriate to address the research question; (b) participant recruitment and data collection were rigorous; (c) the relationship between researcher and participants was adequately considered; (d) ethical issues were addressed; (e) data analysis was sufficiently rigorous; and (f) the findings are clearly stated and valuable. We selected CASP because it is widely recognized in qualitative evidence synthesis, does not impose a single methodological paradigm, and is endorsed by the Cochrane Qualitative and Implementation Methods Group.

Each study was rated as “High credibility,” “Moderate credibility,” or “Low credibility” based on the overall pattern of CASP responses, following the approach recommended by the Cochrane Qualitative and Implementation Methods Group. Studies were not excluded based on critical appraisal; rather, the appraisal results informed the interpretive weighting of findings within the framework synthesis.

#### 2.4.3 Impact of quality assessment on synthesis interpretation

The results of both reporting quality assessment and methodological critical appraisal informed our interpretation of the synthesis findings in two ways:

First, in the framework synthesis, findings from studies rated as high credibility on CASP and with comprehensive reporting on COREQ (≥25/32) were given greater interpretive weight when contradictory or divergent evidence emerged across studies. Findings from studies with significant methodological limitations were not excluded but were interpreted with caution and flagged as potentially less reliable.

Second, common reporting limitations across the evidence base—particularly insufficient description of researcher reflexivity and limited detail on theoretical frameworks—were considered when assessing the overall confidence in review findings. These patterns suggested that some aspects of patient experience may be underreported or influenced by unexamined researcher assumptions.

### 2.5 Data analysis methods

#### 2.5.1 Synthesis approach: best-Fit framework synthesis

This study employed best-fit framework synthesis, as described by Carroll et al. (17, 18). This method is particularly suited to reviews that aim to map patient-reported experiences onto pre-existing conceptual frameworks while allowing for the emergence of novel themes beyond those frameworks.

Framework synthesis was chosen over purely inductive methods (e.g., thematic synthesis, meta-ethnography) because our review question—identifying the outcome domains that patients perceive as meaningful and the contextual factors shaping these experiences—benefits from an organizing structure. The Williamson/Clarke outcome framework (19) (recommended by the COMET initiative) and Marco Testa's contextual factors framework for physical therapy (13) provided initial templates to ensure comprehensive coverage of patient-relevant domains.

However, we explicitly recognize that these frameworks were developed from diverse clinical contexts and may not fully capture the nuances of manual therapy experiences. Therefore, an inductive component was retained to identify emergent themes not accommodated by the *a priori* frameworks. This dual approach ensures that the frameworks organize rather than constrain the synthesis.

#### 2.5.2 Analytical process

The synthesis proceeded through three iterative stages:

Stage 1: Familiarization and Descriptive Coding. Both reviewers (ZY and LSS) independently read each included study multiple times to achieve thorough familiarization. Patient-reported findings were coded descriptively using the original authors' terminology where appropriate. This stage remained “close” to the primary studies. Coding was primarily semantic (staying close to the explicit meaning of the text), with attention to latent meanings where the original authors' interpretations were evident.

Stage 2: Deductive Framework Mapping. Coded findings were mapped onto two *a priori* frameworks:The Williamson/Clarke outcome framework to categorize patient-reported outcome domains;Marco Testa's contextual factors framework to identify environmental and process-related influences on patient experience. Mapping was conducted independently by both reviewers, with discrepancies resolved through discussion. Findings that did not fit within existing framework categories were flagged for inductive analysis.

Stage 3: Inductive Theme Development. Findings that could not be accommodated within the *a priori* frameworks, or that suggested refinement of existing categories, underwent inductive thematic analysis. This involved grouping similar findings, identifying patterns across studies, and generating novel conceptual categories. The balance between deductive and inductive analysis was maintained through iterative discussion among reviewers (ZY, LSS, YCH), ensuring that the *a priori* frameworks organized rather than constrained the synthesis.

#### 2.5.3 Handling of different qualitative methodologies

Included studies employed diverse qualitative approaches (e.g., phenomenology, grounded theory, descriptive qualitative design, thematic analysis). Rather than treating these as interchangeable, we noted each study's methodological orientation during data extraction. Findings from studies with explicit theoretical frameworks (e.g., phenomenological studies emphasizing lived experience) were interpreted within their original epistemological context during synthesis. No study was excluded based on methodological approach; instead, the diversity of approaches was considered a strength that enriched the synthesis.

#### 2.5.4 How the frameworks shaped interpretation

The Williamson/Clarke outcome framework ensured that our synthesis captured the breadth of patient-relevant outcome domains, preventing an overemphasis on biomedical outcomes. Marco Testa's contextual factors framework directed attention to treatment environment, clinician-patient interaction, and organizational factors that might otherwise be overlooked. However, we remained open to findings that challenged or extended these frameworks.

#### 2.5.5 Maintaining balance between deductive and inductive analysis

To prevent the *a priori* frameworks from prematurely closing off emergent themes, we employed the following safeguards:

All coded findings were initially reviewed for “fit” with the frameworks; misfitting data were explicitly documented rather than discarded; Regular team discussions examined whether framework categories adequately captured the data or whether new categories were needed; The inductive stage was conducted independently of the deductive mapping before integration; Novel themes were defined as those that could not be meaningfully accommodated within existing framework categories even after category refinement.

#### 2.5.6 Reflexivity

The research team comprised manual therapy researchers (ZY, LSS, YCH) with clinical experience in manual therapy and training in qualitative methodology. We acknowledge that our professional backgrounds may predispose us to particular interpretations of patient experiences. To mitigate this, we:

Documented analytical decisions, assumptions, and moments of disagreement; Engaged in peer debriefing with a qualitative methodologist external to the clinical context; Distinguished between patient-reported findings and our own interpretive inferences in the presentation of results; Acknowledged our prior assumptions about manual therapy: (a) that patient experience is shaped by multiple biopsychosocial factors, (b) that the therapeutic relationship is important, and (c) that manual therapy may have outcomes beyond symptomatic relief. These assumptions were bracketed during analysis but informed the selection of the *a priori* frameworks.

#### 2.5.7 Relationship between study quality and synthesis inclusion

Consistent with established guidance for qualitative evidence synthesis, we did not exclude studies based on quality assessment results. The rationale for this approach is twofold:

First, qualitative research quality is not uniformly defined across methodological paradigms; criteria appropriate for one approach may be irrelevant for another. Excluding studies based on a single quality framework risks eliminating valuable perspectives from underrepresented populations or novel methodological approaches.

Second, in framework synthesis, the analytical process involves mapping reported findings onto conceptual categories rather than aggregating effect sizes. Therefore, the inclusion of studies with varying reporting transparency does not compromise the synthesis in the same way that including methodologically flawed randomized trials might compromise a meta-analysis. Even studies with limited methodological detail may contribute meaningful patient-reported experiences that enrich the conceptual model.

#### 2.5.8 Protocol and registration

This review was conducted without a pre-registered protocol. Although protocol registration is increasingly recommended for systematic reviews, the exploratory nature of this framework synthesis and the absence of a pre-specified hypothesis rendered protocol registration less critical than for reviews with confirmatory aims. We acknowledge this as a methodological limitation and have ensured transparency by documenting all analytical decisions and providing comprehensive reporting of search strategies, inclusion criteria, and synthesis methods. Future updates of this review will be prospectively registered.

## 3 Results

### 3.1 Results of the literature search

**Figure 1:**
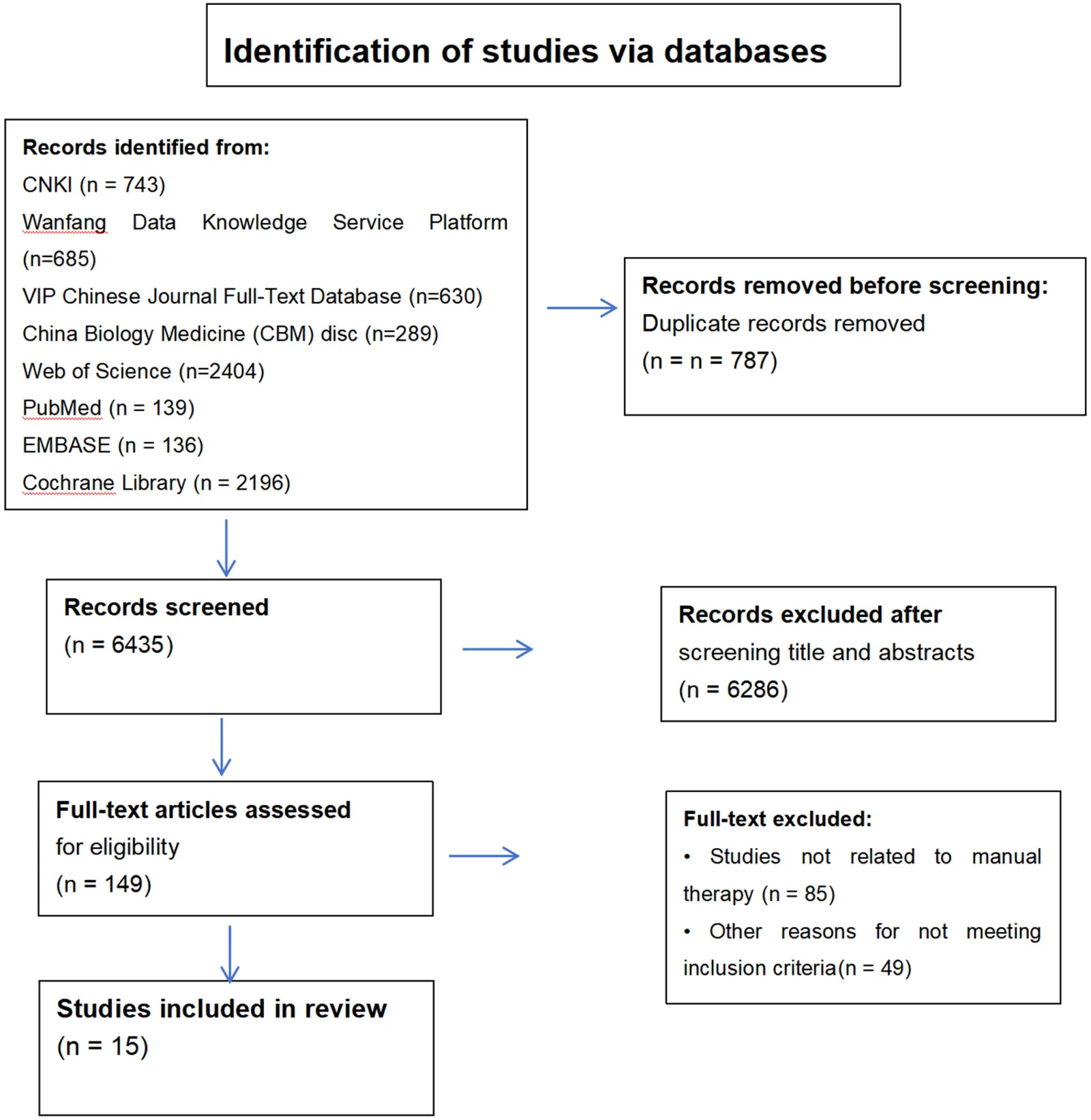
PRISMA flow diagram.

15 articles were finally included in the study (12, 14, 20–32) (Figure 1).

### 3.2 Basic characteristics and quality of the included literature

Basic characteristics of the included literature are shown in Table 1, The COREQ results are shown in Table 2, and the CASP results are shown in Table 3.

**Table 1:** Study characteristics.

Inclusion study	Date of publication	Related field	Research object	The phenomenon of interest	Research type collection methods	Main result
Alfonso-Mora et al. (20)	2023	Tuina	61 patients	Changes before and after the intervention treatment of Mezieres method for low back pain and the overall perception of the treatment received	Mixed study	4 main themes: Pain relief, activities of daily living, quality of life, balance and release of tension
Banton et al. (21)	2023	Bone Setting	Four orthopedic surgeons and four patients	Experience with the phenomenon of cranial bone disease	Phenomenological semi-structural interview	Three main themes: Understanding the framework of skull bone disease, understanding the pathogenesis of skull bone disease, and viewing skull relationships as meaningful rapport
Arnold et al. (22)	2020	Tuina	There were 57 outpatients with depression	Effect of psychoactive massage on outpatients with depression	Mixed research semi-structured interview	3 main themes: Better body awareness and deep relaxation, interruption of rumination and negative thoughts, increased motivation for activities of daily living
Garakyaraghi et al. (12)	2014	Tuina	9 women with prehypertension	The experience of hypertension patients receiving massage	Qualitative research phenomenology in-depth interview	6 main themes: Relaxation, sleeping better, reducing anxiety and tension, reducing fatigue, uplifting the experience, and improving connection
Berger et al. (23)	2019	Tuina	14 patients	Daily life experiences of women with primary dysmenorrhea and their physical perception of dysmenorrhea related symptoms associated with treatment procedures	Mixed research interview	5 main themes: pain, limitations of daily life, body image, body temperature, emotions, and social effects
Steel et al. (24)	2018	Bone Setting	16 patients	Perceptions and experiences of cancer patients receiving osteopathic therapy as a complementary therapy when used in addition to conventional treatment for cancer pain	Qualitative research semi-structured interview rooted theory	4 main themes: Low awareness and high misunderstanding of osteopathy among participants, more than just manipulation, the benefits of osteopathy for cancer-related health complaints, and enabling the continued use of osteopathy
Plank et al. (14)	2021	Tuina	10 patients	Background Expectations and views of manual therapy techniques in patients with chronic low back pain	Qualitative research semi-structured interview	4 main themes: Understanding pain, forming expectations, perceiving care, reassessing body awareness, and management
Anthony Dos Santos et al. (25)	2022	Tuina	Shiatsu user	Motivation and experience in using shiatsu	Qualitative research semi-structured interview	Three main themes: Motivation for using shiatsu (prior knowledge and representations of shiatsu, symptoms leading to its use, ineffectiveness of traditional medicine, barriers to its use) The health trajectory of the user, and the role of shiatsu in the health of the user
Domingos et al. (26)	2014	Tuina	Twenty-two participants were diagnosed with a personality disorder	Significance of aromatherapy massage intervention on mental health of inpatients in psychiatric department	Qualitative research semi-structured interview	2 main themes: Identifying the benefits of aromatherapy and achieving self-awareness
Dikkers et al. (27)	2016	Tuina	13 general practitioners, 10 physical therapists, 7 manual therapists, and 27 patients with neck pain	Barriers and contributing factors affecting the implementation of manual therapy in neck pain management in primary care	Qualitative study Semi-structured interview focus groups	3 main themes: Perceptions of manipulative treatment for neck pain, trust in specific practitioners, background factors of manipulative treatment availability
Ho et al. (28)	2017	Tuina	Fifteen women had cancer	Experience with aromatherapy massage in adult female cancer patients	Qualitative research semi-structured interview	The four main themes are: the immediate effect of bringing all-round comfort and reconnection to everyday life, pleasant moments of forgetting illness with aroma as a booster, the pamper experience of being cared for and maintaining a sense of dignity, and communicating with a failing body
Anne Kelemen et al. (29)	2020	Tuina	20 patients	Perceptions and experiences of inpatient palliative care patients receiving massage therapy from specially trained massage therapists	Qualitative research is rooted in theory	2 main themes: Respondents’ feelings about being in the hospital (invasions of usual care, unconventional treatments, loneliness and loneliness) Respondents’ reflections on the impact of massage on the hospital experience (providing a sense of peace, improving sleep, enhancing relationships, improving coping, physical contact)
Eindhoven et al. (30)	2022	Spinal Adjustment	18 patients	Athletes’ expectations and experiences of sports chiropractor care, and their views on future related research areas	Qualitative research interviews	3 main themes: “I expect no pain”: Athletes’ expectations of sports Chiropractors, “Seeing the whole Picture”: Athletes’ experience with sports chiropractors, “Finding that one in a thousand is not trivial”: Recommendations for the research agenda
Ather Ali et al. (31)	2017	Tuina	18 patients	Participants experienced massage and osteoarthritis	Qualitative study interview	3 main themes: relaxation effects, improving quality of life associated with receiving massage therapy, and accessibility of massage therapy in the treatment of osteoarthritis
Maiers et al. (32)	2022	Spinal Adjustment	50 participants	A view of spinal disability in older adults on the pros and cons of chiropractic therapy and exercise for spine-related outcomes	Mixed research interview	3 main themes: changes in pain, overall sense of improvement, and improvement in biomechanical function

**Table 2:** Assessment of methodological quality of included studies.

Inclusion study/question number	Alfonso-Mora et al. (20)	Banton et al. (21)	Arnold et al. (22)	Garakyaraghi et al. (12)	Berger et al. (23)	Steel et al. (24)	Plank et al. (14)	Anthony Dos Santos et al. (25)	Domingos et al. (26)	Dikkers et al. (27)	Ho et al. (28)	Anne Kelemen et al. (29)	Eindhoven et al. (30)	Ather Ali et al. (31)	Maiers et al. (32)
1	Yes	Yes	No	No	No	Yes	Yes	Yes	Yes	Yes	Yes	Yes	Yes	Yes	Yes
2	No	No	No	No	No	No	Yes	No	No	No	No	No	No	No	Yes
3	Yes	Yes	Yes	Yes	Yes	Yes	Yes	Yes	No	Yes	Yes	Yes	Yes	No	Yes
4	No	No	No	No	No	Yes	No	No	No	No	No	No	No	No	No
5	Yes	Yes	Yes	No	No	Yes	Yes	Yes	Yes	Yes	Yes	No	Yes	No	No
6	No	No	No	No	No	No	Yes	Yes	No	Yes	No	No	No	No	Yes
7	No	No	No	No	No	No	Yes	Yes	No	No	No	No	Yes	No	Yes
8	No	Yes	No	No	No	No	Yes	No	No	No	No	No	No	No	No
9	Yes	Yes	Yes	Yes	Yes	Yes	Yes	Yes	Yes	Yes	Yes	Yes	Yes	Yes	Yes
10	Yes	Yes	Yes	Yes	Yes	Yes	Yes	Yes	Yes	Yes	Yes	Yes	Yes	Yes	Yes
11	No	Yes	Yes	No	Yes	Yes	Yes	Yes	Yes	Yes	Yes	Yes	Yes	Yes	Yes
12	Yes	Yes	Yes	Yes	Yes	Yes	Yes	Yes	Yes	Yes	Yes	Yes	Yes	Yes	Yes
13	Yes	No	Yes	No	No	Yes	Yes	No	No	No	No	Yes	Yes	No	No
14	Yes	Yes	Yes	Yes	Yes	Yes	Yes	Yes	Yes	Yes	Yes	Yes	Yes	Yes	Yes
15	No	No	No	No	No	No	No	No	No	No	No	No	No	No	No
16	Yes	Yes	Yes	Yes	Yes	Yes	Yes	Yes	Yes	Yes	Yes	Yes	Yes	Yes	Yes
17	Yes	Yes	Yes	Yes	Yes	Yes	Yes	Yes	Yes	Yes	Yes	Yes	Yes	Yes	Yes
18	Yes	No	No	No	No	No	No	No	Yes	No	No	No	No	No	No
19	Yes	Yes	No	Yes	Yes	Yes	Yes	Yes	Yes	Yes	Yes	Yes	Yes	No	Yes
20	No	Yes	No	No	No	No	No	No	No	No	No	No	Yes	Yes	No
21	Yes	Yes	No	Yes	Yes	Yes	Yes	Yes	No	Yes	Yes	Yes	Yes	No	No
22	Yes	No	No	Yes	No	Yes	No	Yes	No	Yes	No	No	Yes	Yes	Yes
23	No	Yes	No	Yes	No	No	No	No	No	No	No	No	Yes	No	No
24	Yes	Yes	No	No	No	Yes	Yes	Yes	No	Yes	Yes	Yes	Yes	Yes	Yes
25	Yes	Yes	No	No	No	No	No	No	No	No	No	No	No	No	No
26	Yes	Yes	No	Yes	Yes	Yes	Yes	Yes	Yes	Yes	Yes	Yes	Yes	Yes	Yes
27	Yes	No	No	No	Yes	No	Yes	Yes	No	Yes	No	Yes	Yes	No	Yes
28	No	No	No	Yes	No	No	Yes	No	No	No	No	No	No	No	No
29	Yes	Yes	No	Yes	Yes	Yes	Yes	Yes	No	Yes	Yes	Yes	Yes	Yes	Yes
30	Yes	Yes	Yes	Yes	Yes	Yes	Yes	Yes	Yes	Yes	Yes	Yes	Yes	Yes	Yes
31	Yes	Yes	Yes	Yes	Yes	Yes	Yes	Yes	Yes	Yes	Yes	Yes	Yes	Yes	Yes
32	Yes	Yes	Yes	No	Yes	Yes	Yes	Yes	Yes	Yes	Yes	Yes	Yes	No	Yes

**Table 3:** Results of CASP quality appraisal.

No	Auther (Date)	Q1	Q2	Q3	Q4	Q5	Q6	Q7	Q8	Q9	Q10
1	Alfonso-Mora et al. (2023) (20)	Yes	Yes	Yes	Yes	Yes	CT	Yes	Yes	Yes	Yes
2	Banton et al. (2023) (21)	Yes	Yes	Yes	Yes	Yes	Yes	Yes	Yes	Yes	Yes
3	Arnold et al. (2020) (22)	Yes	Yes	Yes	Yes	Yes	CT	Yes	CT	Yes	Yes
4	Garakyaraghi et al. (2014) (12)	Yes	Yes	Yes	Yes	Yes	CT	Yes	Yes	Yes	Yes
5	Berger et al. (2019) (23)	Yes	Yes	Yes	Yes	Yes	CT	Yes	Yes	Yes	Yes
6	Steel et al. (2018) (24)	Yes	Yes	Yes	Yes	Yes	Yes	Yes	Yes	Yes	Yes
7	Plank et al. (2021) (14)	Yes	Yes	Yes	Yes	Yes	Yes	Yes	Yes	Yes	Yes
8	Dos Santos et al. (2022) (25)	Yes	Yes	Yes	Yes	Yes	Yes	Yes	Yes	Yes	Yes
9	Domingos & Braga (2014) (26)	Yes	Yes	Yes	CT	Yes	CT	CT	Yes	Yes	Yes
10	Dikkers et al. (2016) (27)	Yes	Yes	Yes	Yes	Yes	Yes	Yes	Yes	Yes	Yes
11	Ho et al. (2017) (28)	Yes	Yes	Yes	Yes	Yes	Yes	Yes	Yes	Yes	Yes
12	Kelemen et al. (2020) (29)	Yes	Yes	Yes	Yes	Yes	Yes	Yes	Yes	Yes	Yes
13	Eindhoven et al. (2022) (30)	Yes	Yes	Yes	Yes	Yes	Yes	Yes	Yes	Yes	Yes
14	Ali et al. (2016) (31)	Yes	Yes	Yes	Yes	CT	CT	Yes	CT	Yes	Yes
15	Maiers & Salsbury (2022) (32)	Yes	Yes	Yes	Yes	Yes	CT	Yes	Yes	Yes	Yes

Yes, the criterion was met; CT (Can't Tell), insufficient information to determine; No, the criterion was not met.

The reporting quality of 15 included studies was assessed using the 32-item COREQ checklist. The overall reporting quality was moderate to low, with substantial variation across domains.

Domain 1: Research Team and Reflexivity—Reporting was notably weak. Items related to researcher characteristics (Q2: 13.3% met), relationship with participants (Q4: 6.7%; Q6: 26.7%; Q7: 26.7%; Q8: 13.3%), and reflexivity (Q15: 0%; Q18: 13.3%) were poorly addressed across most studies. Notably, all 15 studies failed to report Q15, indicating a critical gap in researcher reflexivity and self-awareness.

Domain 2: Study Design—Reporting was generally adequate. Theoretical framework (Q3: 86.7%), participant selection (Q5: 66.7%; Q11: 86.7%), and data collection methods (Q9–Q10, Q12, Q14: 100%) were well-reported. However, description of the setting (Q13: 40%) and participant characteristics (Q16–Q17: 100%) showed mixed results.

Domain 3: Analysis and Findings—Reporting was inconsistent. While data analysis process (Q19: 86.7%), presentation of findings (Q21: 68.8%), and use of quotations (Q26: 93.3%) were reasonably well-reported, items related to data saturation (Q20: 20%; Q23: 20%), participant checking (Q22: 50%; Q24: 68.8%), and negative case analysis (Q25: 13.3%) were poorly addressed. Clarity of findings (Q27: 50%; Q28: 13.3%) and researcher contribution (Q29: 86.7%; Q30–Q31: 100%) showed divergent patterns.

Although basic methodological and ethical reporting (Q9–Q10, Q12, Q14, Q16–Q17, Q30–Q31) was generally adequate, critical gaps remain in researcher reflexivity (Q15), researcher-participant relationship transparency (Q2, Q4, Q6–Q8), and analytical rigor (Q20, Q23, Q25, Q28).

The methodological quality of the 15 included studies was generally good. Eight studies met all 10 CASP criteria (rated “Yes” across all items). Six studies had 1–3 items rated as “Can't Tell” (CT), mainly in Q6 (whether the relationship between the researcher and the participants was considered, and whether the researcher adequately reflected) and Q8 (whether the data analysis was sufficiently rigorous). No study received a “No” rating for any criterion.

### 3.3 Outcomes of manual therapy and their influencing factors

Results: A total of 15 studies were included, identifying 2 main themes and 11 sub-themes: Outcome features (clinical symptoms, physical function, emotional function, quality of life, relationship improvement, adverse events); contextual factors (Therapist features, Patient's features, The patient-therapist relationship, Treatment features, Healthcare setting features)

#### 3.3.1 Theme 1: outcome features

The majority of articles report beneficial effects associated with manual therapy interventions. Post-treatment, patients reported improvements in disease symptoms, physical functioning, emotional functioning, quality of life, and social relationships, among other areas.

##### 3.3.2.1 Category 1: clinical symptoms

Participants generally reported that they experienced relaxation, relief from pain and fatigue, improved sleep quality, and improvements in other clinical symptoms in connection with manual therapy.

The relaxation effect of manual therapy interventions was frequently reported by participants, influencing their cognition, affect, and motor activity (12, 20–22, 24, 28, 31).

Massage created a peaceful atmosphere, allowing patients to temporarily forget daily worries and stress: “*Extremely relaxing, I could forget everything. It induced a dreamlike state. I didn't want it to end. I felt wonderful. It was surprising*… ” (12).

“*The massage was unexpectedly relaxing, helping me unwind in such a short time. I definitely felt better, and it reduced some anxiety. It stimulated relaxation*…” (12).

“*Massage therapy was excellent, very soothing, extremely relaxing.*” (31).

Pain relief was consistently reported by participants as a benefit of manual therapy interventions (12, 20–23, 26, 28, 32).

Older adults expressed that symptom management—particularly pain control—constitutes a primary consideration when addressing spinal conditions, which manual therapy can effectively address.

The Mézières method effectively alleviated their low back pain, as described in the participant's account: “*Rising from bed became easier, without the previously experienced excruciating back pain upon awakening*…” (20).

Participants with dysmenorrhea reported pain reductions associated with Rhythmic Massage (RM). Some described progressive monthly improvements in pain, while others reported that radiating back and leg pain had resolved, with only residual mild lower abdominal discomfort remaining. A further subset reported unchanged pain distribution but decreased intensity and shortened duration (23).

Participants reported improvements in pain and paresthesia, reductions in muscle tension and lymphedema, and enhancements in vitality, appetite, and affect associated with aromatherapeutic massage (ATM). Representative accounts documented these effects: “*My pain resolved post-ATM—an unexpected outcome given pre-treatment expectations. Pain intensity decreased from 8 to 2–3 on the numerical rating scale (NRS). I experienced significant physical relief; one participant described: ‘My body felt as though injected with lubricant, enhancing flexibility.’ Others reported increased relaxation and confidence, with one noting: 'Social anxiety diminished—I now engage in community activities without fear, achieving functional restoration comparable to other individuals*.’” (28).

Patients reported improvements in cancer-related pain and other symptoms either immediately or over time following osteopathic treatment, including constipation, fatigue, circulation, and edema: “*Right after treatment, within a day or two, I was indeed tired, but definitely experienced less pain*… *Now I can get up daily, and yesterday I even ran, something I hadn't done in a long time.*” (24).

Participants consistently reported that improvements in sleep and reductions in fatigue were particularly important or meaningful to them.

Female participants reported improved sleep quality associated with therapeutic massage. Participants reported that therapeutic massage was associated with reduced anxiety, relaxation, and improved sleep: “*After falling ill, I couldn't sleep for a long time. I checked the clock every hour throughout the night, waiting for time to pass. Since receiving massage therapy last week, I've finally been able to sleep well—something I hadn't managed for weeks. The massage helped me relax, enabling me to get proper rest*…” (12).

Participants with depression reported finding psychological massage useful in daily life as a tool associated with improved sleep (22). Among 22 participants in aromatherapy studies, 20 individuals (91%) reported improved sleep patterns and reduced difficulty falling asleep. Participants reported that sleep improvements occurred almost immediately after the initial intervention session (26).

Female participants reported heightened energy levels associated with therapeutic massage. They reported resolution of fatigue and described massage as providing time for rest and relief from physical weakness, exhaustion, and intrusive thoughts. They suggested that these experiences were associated with stimulated energy flow and alleviation of psychosomatic fatigue: “*This is amazing—I don’t feel tired after the massage. I’m fully energized, with no residual fatigue. I can fulfill my responsibilities, and interestingly, all my daily fatigue vanished with the massage*…” (12).

“Massage was enjoyable. I felt all my daily fatigue dissipate completely, experienced sensations I had never felt before, and it gave me energy. I sensed my body operating in a different way. Now I feel I can do anything I want to…” (12).

##### 3.3.2.2 Category 2: physical function

Patients consistently reported enhanced bodily awareness associated with manual therapy interventions. Participants described changes in physical function in terms of reduced restrictions in daily activities and altered bodily perception.

Participants reported viewing the Mézières method as a process associated with learning better body control, improved posture, and reduced pain. Through practicing specific postures and movements, participants reported improved understanding of bodily sensations and movement, and described being better able to identify and correct poor posture. They reported: “*Hey, I haven't experienced those terrible moments of feeling stuck anymore, unable to drive or walk like I used to, or feeling unable to turn my head because I was stuck. I haven't experienced those moments.*” “*I also notice postural changes, especially at the neck and back level, with improved postural alignment. I'm much more aware of my posture now*… ” “…*Previously, when I take a shower and apply lotion, I couldn't bend to put lotion on my legs because it was very painful. I felt stiff. I lacked the ability to bend*… *Now I feel more flexible.*” (20).

Participants reported enhanced somatic awareness associated with therapeutic interventions, and described greater self-acceptance related to changes in bodily perception. For instance, women who received Rhythmic Massage (RM) for dysmenorrhea reported experiences of profound somatic reorganization, including heightened recognition of physiological boundaries and improved thermosensory integration. As documented: “*I perceive my body as more integrated*… *experiencing warmth in my feet during massage. From session to session, thermal equilibrium becomes more sustained*” (23).

Similarly, among 14 cancer patients, aromatherapeutic massage (ATM) mediated therapeutic touch that transformed bodily relationships. Participants described enhanced capacity to accept anatomical alterations post-surgery, with one participant reporting: “*The extensive axillary scar left me with tactile avoidance. Light touch evoked no sensation—I perceived this area as numbed. Yet during ATM, sensation returned. That epiphany revealed how I'd avoided viewing or touching my breast since surgery. This neglected region requires reconnection: I must communicate with my body through deliberate tactile engagement.*” (28).

##### 3.3.2.3 Category 3: emotional function

Participants reported emotional and social impacts associated with manual therapy interventions, including reduced negative affect, enhanced self-awareness, and improved disease coping capacity (12, 22, 23, 26, 28).

Aromatherapeutic massage combined manual techniques and essential oils to generate somatic stimulation and pleasurable experiences, facilitating respite from illness-induced vulnerability (28).

Patients receiving psychotherapeutic massage described compassionate professional touch penetrating communication barriers to access dysregulated emotional states:

“…I regained sensory awareness in my legs and feet… Never had I experienced such loving touch. Depressive avoidance previously prevented such contact… My emotional armor softened, enabling psychosomatic reintegration. The therapist’s attuned touch restored my embodied wholeness.” (22).

Women who received therapeutic massage reported reduced anxiety associated with the intervention:

“Remarkably beneficial for tension reduction—this serene intervention should be prescribed for anxiety disorders.” (12)

Several participants described empowerment through treatment:

“Post-treatment vitality restored my agency—both physically and psychologically. This profound control is exhilarating.”

“Effective rejuvenation equivalent to 10–15 years’ reversal, optimizing daily functional capacity.”

“Induced recalibration of self-regulation—an indescribable fortification particularly noticeable on treatment days.” (12)

Aromatherapeutic massage facilitated metacognitive reflection in personality disorder patients, enhancing symptom management. Participants recognized how emotional dysregulation and impulsivity perpetuated relational distress. Through massage-induced reflection, they reconceptualized symptoms as modifiable personality traits (26).

##### 3.3.2.4 Category 4: quality of life

Participants associated manual therapy with quality of life (QoL) improvement (17, 23, 31):

Mézières method users explicitly referenced QoL changes:

“Pain modulation defines QoL… Breathing techniques restore equilibrium—establishing tranquility and functional well-being.” (20)

Participants with dysmenorrhea reported multidimensional improvements associated with rhythmic massage, including reduced pain, enhanced bodily awareness, and improved quality of life (23).

Beyond reported pain relief and functional knee improvement, participants described eudaimonic well-being associated with massage:

“Recontextualized osteoarthritis as manageable rather than debilitating… This pervasive wellness enables joyful activity engagement.” (31)

##### 3.3.2.5 Category 5: improved relationships

Patients reported massage attenuated loneliness and anxiety, facilitating improved familial, spousal, and social connections:

“Enhanced sociability persists post-session—I actively seek interpersonal engagement over isolation.”

“Markedly improved conflict resolution within family dynamics.”

“Treatment days optimize relational functioning across all social spheres.” (12)

##### 3.3.2.6 Category 6: adverse events

Patients expressed safety concerns regarding cervical manipulation techniques (particularly joint cavitation), preferring physiotherapy as primary intervention One participant with prior manual therapy experience stated:

“High-velocity thrust techniques involve uncontrollable risk intensity. My iatrogenic injury required extended recovery—such interventions should remain last-resort options.” (27)

#### 3.3.2 Theme 2: contextual factors

##### 3.3.2.1 Category 1: therapist features

The “prestige effect” exists and influences patient treatment outcomes. Patients prioritize therapists' expertise and reputation, selecting practitioners based on established trust. They collaborate with therapists possessing proven experience in treating specific conditions, obtain referrals from trusted sources, and develop effective therapeutic alliances to enhance treatment efficacy. For instance, clinics housing specialized manual therapists with expertise in particular cervical disorders attract such patients. Patients often select therapists based on receptionists' recommendations: “*I trust my chosen physician. Having resided here long-term with positive interactions, I assumed this doctor would recommend a manual therapist he endorses.*” (27).

All participants receiving sports chiropractic care expressed trust in their practitioners and felt comfortable discussing all aspects of training and personal health: “*He’s the pro here, so there’s immense trust in this relationship. When he says, ‘Hey, this feels tight,’ or ‘This isn't functioning optimally,’ he'd address it immediately—I find this exceptional.*” (30).

##### 3.3.2.2 Category 2: patient's features

Patients' perceptions and direct experiences constitute core elements influencing therapeutic efficacy. Included literature reports patients' motivations, cognitive shifts, and treatment expectations during manual therapy interventions.

###### 3.3.2.2.1 Motivation

Patients sought manual interventions due to intolerable disease symptoms. Initial limited disease awareness or misconceptions about interventions led them to consult general practitioners first, following conventional healthcare pathways. Some discovered medication inefficacy, while others feared pharmacological side effects. Facing conventional medicine's limitations, patients pursued alternatives like acupressure, osteopathy, or massage:

“After a dreadful migraine, I had the opportunity to experience acupressure.”

“Some medications worked but upset my stomach. Ultimately, this allowed me to reduce my medication load.”

“Conventional medicine offered no solutions—my immediate response was seeking non-conventional alternatives.” (25)

###### 3.3.2.2.2 Cognitive Shifts

Patients often lacked understanding of manual interventions' mechanisms prior to participation, sometimes holding misconceptions. Post-treatment experiences reconfigured their perceptions, establishing manual therapy as a comfortable modality. For example, osteopathy-naïve patients reported preconceptions of abrupt manipulations:

“Initially apprehensive—I feared possible maneuvers… Then I realized it differed completely from expectations of forceful twisting. Everything proved exceptionally gentle… truly manipulation-free… thus risk-absent.” (24)

Craniosacral osteopathy recipients initially adhered to mind-body dualism. Intense somatic experiences during treatment transformed pre-treatment pain and fragmentation into post-treatment wholeness, validating the therapy's efficacy:

Joanna: “I have empirical proof! Post-session, I walked straight… beyond my autonomous capacity.”

Eva: “You simply feel integrated—mind-body unity… aware of… internal landscapes… reconnecting feet to earth.” (21)

###### 3.3.2.2.3 Expectations

Treatment expectations formed through prior experiences, social context, and therapist communication. Previous encounters served as primary determinants. Extensive unsuccessful treatments typically lowered expectations. For instance, most patients considered exercise the optimal self-management strategy; consequently, non-exercise techniques (e.g., sham treatment) failed expectations:

“…I perceived null effects—like an inferior embrace! (laughs) Utterly irrelevant per my criteria. No detectable changes occurred.” (14)

Specific recommendations from social networks (friends/family) or positive prior experiences elevated expectations. One participant explained why manipulative techniques outperformed non-manipulative approaches:

“…Likely due to joint cavitation. I habitually associate cavitation with pain relief—like post-exercise adjustments. The audible release signifies resolution.” (14)

Participants held divergent clinical expectations based on treatment histories. Real-time efficacy reassessments occurred during interventions, with post-treatment evaluations influencing technique selection and overall satisfaction while modifying future expectations. Athlete-patients initially expected chiropractors to manage injuries and alleviate symptoms. As treatments progressed, efficacy met expectations, shifting goals from pain resolution to injury prevention and performance enhancement:

“Initially, I only expected pain cessation. After two sessions, I realized I'd emerge not just pain-free, but functionally superior to pre-injury status.” (30)

Non-technical factors also influenced expectations. Patients evaluated treatment quality through perceived therapeutic communication. Effective explanations enhanced comfort and satisfaction:

“…Enhanced dialogue improves treatment quality. Understanding pain origins and intervention mechanisms maximizes therapeutic benefit.” (14)

##### 3.3.2.3 Category 3: the patient-therapist relationship

A positive therapeutic alliance significantly influences treatment outcomes. Clinical communication between practitioners and patients encompasses verbal and nonverbal dimensions.

###### 3.3.2.3.1 Verbal Communication

Appropriate verbal exchange constitutes a prerequisite for effective therapeutic relationships. Manual therapists engage in dialogue throughout the treatment continuum.

Patient satisfaction correlates with perceived technical competence and therapeutic communication. Participants with low back pain receiving manual interventions reported heightened comfort when therapists adapted communication to their knowledge level and interests, utilizing informal dialogue for information exchange:

“…Using simpler language rather than precise terminology benefits those like me with limited medical literacy—complex terms remain incomprehensible.” (14)

“…Oh cool! He’s palpating my L4-L5. Knowing the specific segments being treated matters—other clinicians never identify them.” (14)

Aromatherapeutic massage enhanced perceived autonomy through choice in essential oils and treatment areas:

“Therapist prioritized my identified discomfort zones. Olfactory selection of acceptable essential oils reinforced mutual respect.” (28)

###### 3.3.2.3.2 Nonverbal Communication

The therapeutic relationship significantly impacts treatment efficacy. Beyond verbal exchange, nonverbal communication predominantly conveys respect and care within the clinical encounter.

Craniosacral osteopathy participants identified the practitioner-patient relationship as mechanistically central. Osteopathic communication operates primarily nonverbally, with meaningful experiences transcending verbal description:

Eva described the “wave” sensation enabling profound relaxation: “That…feeling…that’s what relaxation embodies.” The intervention facilitated an innate sense of wellness: “To sense that…something deeper…truly profound…indescribably beautiful—our natural state of being.” (21)

Aromatherapy recipients perceived care and respect through nonverbal interaction:

“Her hands communicated with my body through nurturing touch. This somatic dialogue induced tranquility.” (28)

“Infant-gentle contact characterized the entire session.” (28)

##### 3.3.2.4 Category 4:treatment features

Various therapeutic variables influence patients' perceived outcomes.

###### 3.3.2.4.1 Knowledge Acquisition

Patient acquisition of disease-related knowledge—including clear diagnosis, pathophysiology explanations, intervention rationale, and prevention strategies—significantly impacts satisfaction (21, 24, 26, 30).

Patients reported osteopaths' diagnostic accuracy exceeded expectations. Clear explanations fostered reassurance:

“His approach to pain demystified it. Through discussing causes, he made everything less dramatic.” (24)

Participants valued understanding pain etiology and treatment objectives:

“For pain relief… nobody knew its origin… He precisely identified the source through palpation—'This muscle is responsible.'” (24)

Craniosacral osteopathy recipients initially lacked mechanistic understanding but accepted efficacy through direct experience, developing personal metaphors:

Richard perceived his osteopath “projecting intent through touch to unblock circulatory pathways” (“pulsating internal circulation… like opening a faucet”). Joanna described her therapist as “a counselor affecting mind-body unity,” noting “emotional release” and “morphological changes” signifying pain acceptance (21).

Sports chiropractic participants recommended research on injury mechanisms/prevention and performance enhancement:

“Understanding how lifting posture affects the back could prevent issues before they start.” (30)

“Determining when training intensity becomes counterproductive—when the body breaks down tendons rather than building muscle.” (30)

###### 3.3.2.4.2 Patient-Centered Approach and Holistic Care Process

Treatment holism, patient consideration, organizational factors, and patient-centeredness influence outcomes.

##### 3.3.2.5 Treatment holism

Manual therapy inherently addresses the whole person (24, 25, 30). Osteopathy patients experienced comprehensive care:

“Beneficial for the entire body—not just isolated parts.” (24)

Osteopaths dedicate extensive initial consultations incorporating manual techniques, lifestyle advice, and patient education.

Sports chiropractic assessments examined multiple body regions beyond primary complaints:

“She conducted thorough baseline assessments—seeing the whole picture to optimize treatment rather than spot-fixing issues.” (30)

##### 3.3.2.6 Patient-Centered therapeutic alliance

Literature consistently emphasizes patient-centered relationships (21, 28–30, 32).

Manual therapy patients established strong therapeutic bonds:

“I trust Dr. X completely—his deep understanding of my injury history ensures continuity of care.” (30)

Massage recipients valued empathy and active listening:

“Feeling genuinely cared for by practitioners who believe in their work.” (29)

Metaphorical language in craniosacral therapy fostered shared understanding:

Richard described restored leg circulation: “My leg has truly returned.” (21)

Chiropractic patients highlighted relational elements:

“Everyone needs to feel cared for—from receptionists to doctors, this team shows authentic concern.” (32)

Aromatherapy participants noted personalized environmental adjustments:

“Every detail—heated blankets, temperature, lighting—reflected individualized care.” (28)

##### 3.3.2.7 Treatment organization and procedures

Logistical factors affect treatment accessibility and timing.

Osteopathy's extended consultations facilitated patient education and non-pharmacological pain management (24).

Sports chiropractic patients valued sufficient time for comprehensive discussion:

“Longer appointments ensured all concerns were addressed.” (30)

Flexible scheduling was essential for massage accessibility:

“Many lack support systems during hospitalizations.” (29)

Cost barriers limited ongoing access:

“I'd receive regular massage for osteoarthritis if covered by insurance.” (31)

Organizational constraints included:

- Waitlist limitations
- Gender-preference mismatches
- Administrative inefficiencies (27)

###### 3.3.2.7.1 Therapeutic Touch

Tactile interaction regulates social bonding. Clinical touch alleviates musculoskeletal pain.

Literature consistently reports manual therapy's positive perception. Compassionate touch mitigates social isolation. Participants described therapists' hands as inherently healing:

“I wept during treatment—finally feeling my legs through loving touch. Depression had prevented such contact… My emotional armor softened through this gentle, reintegrative experience.” (22)

Some achieved meditative surrender:

“Like meditation… His non-intrusive gentleness induced near-sleep—a tranquil respite from thought.” (24)

##### 3.3.2.8 Category 5: healthcare setting features

Sensory elements within the therapeutic environment modulate patient outcomes. Settings featuring natural lighting, low noise levels, relaxing background music, pleasant aromas, and optimal temperature regulation are consistently preferred.

Patients receiving psychotherapeutic massage in depression clinics reported suboptimal environmental conditions, including:

- Thermal discomfort during disrobing
- Body image discomfort exacerbated by cold settings
- Absence of background music noted as a deficiency
- Noise disruption from adjacent construction sites (22)

ATM synergized tactile stimulation with essential oils to generate pleasurable somatosensory experiences. Participants described enhanced therapeutic effects:

“ATM differs significantly from plain massage—increased smoothness with essential oils serving distinct purposes. Their aromas function as psychological boosters.” (28)

“Physical revitalization occurs alongside mood elevation and energy restoration.” (28)

Environmental warmth conveyed holistic care, facilitating inner peace:

“The ambient comfort was notable—precise temperature modulation with post-session warming blankets. Every element received deliberate consideration: lighting adjustments, thermal inquiries… personalized environmental attunement.” (28)

### 3.4 Manual medicine: the assessment-intervention-feedback diagnostic-therapeutic system and implementation pathway

**Figure 2:**
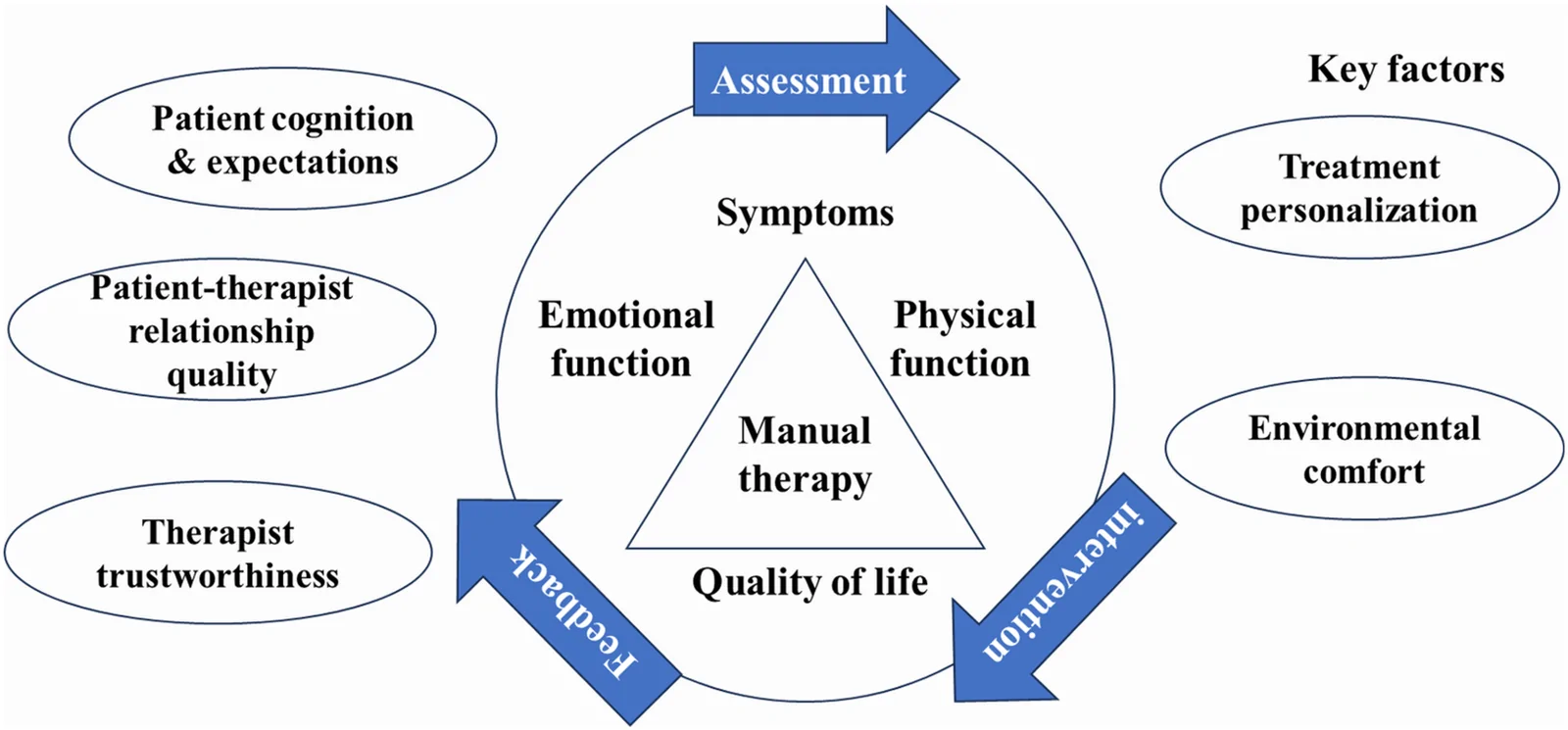
Manual medicine assessment-intervention-feedback diagnostic and treatment system and implementation pathway.

Manual Medicine establishes its foundation upon an “Assessment-Intervention-Feedback” diagnostic-therapeutic closed-loop system, evolving into a distinctive biopsychosocial integrative therapy characterized by tactile mediation, dynamic adaptation, and multisystem synergy (see Figure 2). Its core competitive advantages over conventional medical approaches manifest in four key technical dimensions: the Tactile Information Transmission System, the Dynamic Adaptive Regulation Mechanism, the Multisensory Integration Network, and the Cross-Dimensional Efficacy Assessment System. These elements engage in progressive synergistic interplay throughout the entire diagnostic-therapeutic cycle.

#### 3.4.1 Assessment phase: the tactile-mediated multidimensional perception system

The assessment framework of Manual Medicine is defined by its core reliance on tactile mediation. Therapists utilize manual palpation to establish a dynamic perceptual network encompassing biological, psychological, and social dimensions. As a primary diagnostic tool, tactile perception enables the simultaneous analysis of tissue biomechanical status (e.g., fascial tension, joint range of motion) and patient psychological feedback (e.g., tactile tolerance, emotional stress signals), offering information density far exceeding the unidimensional data obtained through visual inspection or instrumental examination. The dynamic adaptation characteristic is evident in the real-time interpretation of and response to tactile signals. Therapists dynamically adjust assessment strategies based on tissue resistance changes during palpation, generating individualized biomechanical profiles. Concurrently, multisensory integration optimizes tactile sensitivity through environmental design (e.g., lighting, temperature), mitigating patient defensive reactions and ensuring the ecological validity of assessment results. Ultimately, the assessment system is outcome-oriented, integrating indicators such as pain intensity, affective responses, and social function to construct a multidimensional baseline model that transcends traditional biomedical frameworks.

#### 3.4.2 Intervention phase: precision modulation through dynamic adaptation and multisensory synergy

The core of the intervention phase lies in the dynamic adaptation mechanism. Through tactile-mediated real-time feedback loops, millisecond-level fine-tuning of manual parameters (pressure, frequency, direction) is achieved. The therapist's tactile perception functions dually as both the intervention medium and the regulatory sensor, dynamically optimizing the intervention trajectory based on tissue biomechanical responses (e.g., viscoelastic changes) and patient physiological signals (e.g., EMG activity). Multisensory integration further amplifies intervention efficacy: environmental temperature modulates tactile receptor sensitivity; olfactory stimuli (e.g., essential oil molecules) enhance emotional regulation via the limbic system; auditory guidance (e.g., breathing rhythm cues) synchronizes neuromotor control. This multimodal synergy overcomes the limitations of unimodal sensory interventions, creating a resonance effect between mechanical stimulation and neuromodulation. The intervention process consistently targets multidimensional outcomes, concurrently promoting synergistic improvements in pain relief (biological), anxiety suppression (psychological), and social function activation (social), demonstrating Manual Medicine's holistic mastery over complex systems.

#### 3.4.3 Feedback phase: multidimensional outcome verification and system iteration

The feedback phase establishes a closed-loop optimization system for manual interventions through the quantitative verification of multidimensional outcomes. Biological markers (e.g., pain scores, joint range of motion), psychological scales (e.g., anxiety indices, self-efficacy), and social function parameters (e.g., interpersonal relationship satisfaction) collectively form a three-dimensional evaluation matrix, objectively reflecting the cross-dimensional benefits of the intervention. The dynamic adaptation characteristic manifests here as data-driven strategy iteration: machine learning algorithms parse efficacy-factor correlations to dynamically adjust subsequent intervention parameters (e.g., pressure intensity, treatment frequency). The tactile modality extends into remote follow-up, with wearable devices enabling continuous monitoring of patient somatosensory feedback, forming a long-term efficacy tracking network. Simultaneously, environmental modulation data from multisensory integration (e.g., treatment space temperature/humidity records) are incorporated into the analysis, revealing the modulatory patterns of exogenous factors on therapeutic outcomes. This feedback system not only validates the multidimensional value of Manual Medicine but also propels its paradigm shift from experiential art towards precision science.

Through its chained operational framework of “Tactile Mediation—Dynamic Adaptation—Multisensory Integration—Multidimensional Outcomes,” Manual Medicine achieves synergistic regulation of the biopsychosocial system across the assessment, intervention, and feedback phases. Its core characteristics are mutually embedded and dynamically interwoven, collectively constructing a unique interventional paradigm distinct from pharmacological, surgical, and other physical therapies.

## 4 Discussion

### 4.1 Main findings

This synthesis of 15 qualitative studies suggests that manual treatments (such as massage, osteopathic treatment, and craniosacral therapy) are associated with multifaceted health outcomes, and identifies factors influencing these reported experiences. Participants reported improvements in quality of life, physical function (mobility, postural control), emotional function (anxiety relief, self-acceptance), and clinical symptoms (e.g., pain, fatigue, sleep disturbances) associated with manual therapies, though they also raised questions about the safety of medical procedures. Trustworthiness of the therapist, patient expectations and cognition, the quality of the patient-therapist connection, treatment individualization, and comfort in the environment were identified as key factors influencing participants' experiences of manual therapy.

This study, for the first time, explains how manual medicine achieves synergistic regulation of the biological-psychological-social system across the three phases of assessment, intervention, and feedback through a chain-like action framework of “tactile medium- dynamic adaptation- multisensory integration- multidimensional outcomes,” by integrating qualitative evidence. It provides a theoretical foundation for the formulation of health policies as well as clinical practice in evidence-based complementary medicine.

### 4.2 Multidimensional characteristics of manual therapy intervention

These reported outcomes—including clinical symptoms, quality of life, relationships, physical and emotional function, and adverse events—align with the biopsychosocial medical paradigm. This model proposes that the dynamic interplay of physiological, psychological, and social elements influences health and disease, offering a broader perspective than one-dimensional biological approaches. Proposed mechanisms suggest that manual therapy may be associated with improved motor performance in pain patients through modulation of fascial tension, as well as changes in cortisol levels and sympathetic nervous system activity. These physiological changes may contribute to reduced anxiety and pain catastrophizing, and improved social function through greater social involvement (30–33). Empirical studies indicate that manual therapy is associated with improvements in anxiety and quality of life, and reductions in kinesiophobia and pain catastrophizing, in patients with lumbar disc herniation (10). Additionally, findings from “Wo Hu Shi” (Crouching Tiger Pose) training suggest a connection between biological, psychological, and social outcomes, as indicated by increased multifidus muscle activity and improved SF-36 emotional scores in patients with non-specific low back pain (34).

Proposed mechanisms suggest that manual therapy may exert synergistic effects at biological, psychological, and social levels. Findings from (35) suggest that manual treatment may engage endogenous analgesic mechanisms through descending regulatory pathways, such as modulation of inhibitory interneurons in the spinal dorsal horn. Additionally, data from (36, 37) indicate that different manual methods may be associated with modified transmission of pain signals through effects on different nerve fiber types. Hypothesized mechanisms further suggest that manual therapy may be associated with changes in neurotransmitter release, including increased oxytocin and decreased cortisol, which may stimulate parasympathetic nerve activity. These changes may be associated with improved blood circulation, relaxation, sleep-wake cycle regulation, and reduced fatigue (38–40). Findings from (41, 42) suggest that tactile input may be associated with improved physical function through neuroplastic changes in the primary somatosensory cortex (S1). Additionally, proposed mechanisms suggest that fascial tension management may be associated with changes in whole-body mechanical balance through altered body perception via tactile-proprioceptive integration, described as a “mechanical-neural coupling” process (43, 44). Data from (45) indicate that manual therapy may be associated with improved mood through changes in serotonin, dopamine, and cortisol levels.

The smooth flow of blood and qi is frequently associated with delicate tactile stimulation in Eastern cultures. Treatments like as tuina and gentle massage are said to improve circulation, calm the mind, and control internal organs. This aligns with the idea that gentle tactile input activates the brain's reward system, releasing neurotransmitters such as dopamine to induce a sense of physical and mental comfort. Manual therapy can improve relationships and quality of life by releasing oxytocin from C-tactile fibers, which promotes relaxation and a sense of security. This strengthens bonds and trust in interpersonal relationships and has a positive impact on mental health and emotional stability (46). Additionally, tactile-mediated emotional attachment extends the “care bond” between practitioners and patients to family relationships.

Both physiological and cultural aspects must be taken into account when assessing the risks of adverse outcomes associated with manual treatment. The vertebral artery may experience shear stresses as a result of high-velocity low-amplitude (HVLA) manipulation of the cervical spine, according to biomechanical theory. Yet, research shows that when cervical HVLA manipulation is done by experts, the strain is typically less than that from regular neck motions (such as looking up or turning the head in the same direction), which makes mechanical damage to the vertebral artery unlikely (47). To reduce possible dangers, a comprehensive preoperative evaluation of patients undergoing cervical HVLA manipulation remains necessary, especially for those with risk factors for vertebral artery disease.

Therefore, to obtain more thorough and successful therapeutic outcomes, both the physiological dangers of the technique and its beneficial psychological and emotional effects via tactile stimulation should be taken into account in the risk assessment and implementation of manual therapy.

### 4.3 Multifaceted contextual factor regulatory mechanisms

This research discovered that the contextual impact system of manual therapy interventions is made up of the following elements: practitioner professional competency, patient individual characteristics, quality of practitioner-patient interaction, treatment method features, and medical setting design. This system has a major impact on clinical outcomes by controlling the synergistic connections of biopsychosocial pathways.

#### 4.3.1 Practitioner qualities: core competitiveness driven by tactile skills

A fundamental component of manual therapy is tactile skills. The technical competency of professionals is demonstrated in clinical practice by their ability to do soft tissue palpation, joint mobilization, show empathy in practitioner-patient interactions, and provide a safe atmosphere (48). Experts are able to precisely detect anomalies in tissues and joints and treat patients by referring to technical guidelines and customizing care for each patient's needs. In manual therapy, touching produces positive outcomes in a number of areas, such as somatic perception, emotional communication, and analgesia. The physical characteristics of stimulation, such as position, intensity, and frequency, as well as the emotional qualities that come from a caring patient-practitioner connection, are how practitioners' manual approaches work. This illustrates the comprehensive knowledge of anatomy, biomechanics, and neurophysiology that manual therapists possess (48, 49). Touch has been shown to improve patients' somatic perception, their sensation of control (“I control my body”), and their sense of ownership (“This is my body”). The primary competitive advantage of manual therapy is its tactile dynamic adaptation capability, which allows for real-time perception of tissue mechanical changes while communicating humanistic care through tactile feedback. This is in contrast to psychological counseling, which depends on verbal empathy, or surgery, which depends on instrumental precision. This two-pronged benefit of “technique-empathy” has multifaceted biopsychosocial outcomes.

#### 4.3.2 Patient features: somatic experiences mediating the efficacy bridge

Expectations have a significant role in controlling patients' mental, emotional, and physical experiences. Verbal recommendations are the easiest and most direct way to build these expectations, which are constantly updated and changed by inputs from the environment. Compared to neutral suggestions, positive verbal suggestions can relieve mechanical hyperalgesia and pain intensity in patients with neck pain, and patients report much less discomfort (50). On the other hand, a “nocebo effect” may occur when patients sense dread as a result of negative social information or past unpleasant experiences (e.g., “manual therapy may exacerbate the condition”), which may intensify pain perception through the prostaglandin pathway (51). Unlike pharmaceutical treatments, which rely on pharmacological information to form expectancies, manual therapy's success is more dependent on embodied experience—tactile input immediately corrects pain-catastrophizing cognition. Manual therapy has a clear embodied advantage over virtual reality treatment, as its tactile-proprioceptive integration speeds up the restoration of somatosensory maps (52). Furthermore, patients' treatment preferences and historical experiences might influence the amount of treatment response during rehabilitation, as they compare their present findings to previous positive or negative results. Treatment results may suffer if patients' preferences, expectations, and past experiences are ignored. As a result, patients' tactile perceptions function as brain interfaces that link biological processes and psychological cognition in addition to being carriers of therapeutic efficacy.

#### 4.3.3 Relationship between patient and practitioner: neurological effectiveness of tactile empathy transmission

Good interactions between patients and practitioners reduce sympathetic nerve excitability, which improves physiological signs like pain. Relationships between patients and clinicians are governed by both verbal and nonverbal cues in therapeutic settings. Patient satisfaction and treatment results are impacted by appropriate verbal communication, which includes active listening, promoting verbal expressions, and respecting patients' viewpoints. Positive statements about pain reduction (such as “This treatment is a potent analgesic”) have been shown in medicine to provide greater analgesic effects than placebos. Conversely, negative emotions arising from patients perceiving the practitioner as indifferent or impatient may amplify pain perception through neuroendocrine pathways (such as the CCK pathway), negating the biomechanical benefits of manual treatment (53–55). The effectiveness of treatment is also influenced by nonverbal cues used in therapeutic settings, such as smiling and maintaining eye contact.

Studies demonstrated that patients can use nonverbal clues to deduce the intentions of practitioners, which activates the prefrontal-limbic system, causes them to subconsciously change their own behaviors, and improves their sense of safety and treatment compliance (56, 57). Manual therapy, as opposed to general medical treatment, which is based on medical history, or “hands-off” therapies that lack emotional support, improves the predictability of therapeutic success by establishing tactile trust.

#### 4.3.4 Treatment features: A patient-centered strategy and multisensory collaboration

The patient is at the heart of the whole manual diagnostic and treatment procedure. A key component of making patients satisfied with the practitioner during consultations is giving them thorough explanations of the disease's background, diagnosis, treatment strategy, and prevention. Second, patient satisfaction is increased by the entire treatment process arrangement's rationality as well as by taking patients' viewpoints into account. Furthermore, manual therapy, as a specialized type of touch, when combined with verbal communication, as well as the use of essential oils, allows for multisensory synergy to enhance therapeutic efficacy. Studies have demonstrated that multi-modal integration of sensory stimuli (e.g., olfactory, tactile, and auditory) can enhance physiological and psychological recovery effects when compared to single sensory stimulation. This is because combinations of different sensory stimuli may activate distinct neural pathways and brain regions (58). The “patient-centered” and “multi-modal” features of manual therapy form a precision regulation closed-loop, with its full-chain service and multi-sensory synergy serving as the scientific basis for optimizing therapeutic efficacy.

#### 4.3.5 Features of the medical setting: professional medical space design

Patients' physical and emotional moods might be impacted by the therapeutic environment in the therapy setting. Peaceful music can help patients relax and lessen their stress reaction; the aroma of essential oils can provide a sense of peace and tranquility; and a private quiet setting can help patients feel psychologically comfortable and less ashamed about undressing during treatment. The “ritualistic design of treatment spaces” in professional medical settings can maximize the biopsychosocial benefits of manual therapy interventions by improving their physiological effects and psychological safety in contrast to traditional consultation rooms that prioritize visual privacy and home environments that lack multi-sensory integration.

The determinants of manual therapy's “biopsychosocial” efficacy are meticulously revealed by the following factors: the tactile expertise of practitioners propels accurate treatments; patients' somatic experiences build cognitive bridges; tactile empathy between patients and practitioners fortifies social bonds; dynamic multi-modal design allows for customized adaptation; and tactile-friendly surroundings enhance efficacy through neuroecological mechanisms. This system differs from single-dimensional therapies such as pharmacotherapy and surgery, with its core competitiveness residing in the multi-level dynamic adaptation capability mediated by touch—from millimeter-level mechanical perception to second-level neural feedback, and from individual cognitive reshaping to social relationship repair—reflecting a paradigm shift in integrative medicine.

### 4.4 Manual therapy pathways for implementation of diagnostic and treatment systems

The intricate relationship between neurobiology and biomechanics reflects the fundamental scientific reasoning of this diagnostic and therapeutic approach. The recently identified role of the Piezo2 mechanosensitive ion channel, which transforms mechanical stimuli into electrical signals and directly acts on the dorsal root ganglion-anterior cingulate cortex pathway, is highly consistent with the dynamic perception mechanism mediated by tactile sensation. This provides a molecular biological basis for the synchronous assessment of “tactile-emotion.” The “adaptive control model” from complex systems theory serves as the basis for the dynamic adaptation mechanism at the biomechanical level. Manual treatment settings and tissue responses are dynamically matched through real-time tissue manipulation simulation with ongoing monitoring and feedback.

This strategy has succeeded in achieving a paradigm shift at the level of scientific construction from conventional empirical medicine to data-driven medicine. Its therapeutic reasoning is evident in the physiological adaptation of multidimensional intervention mechanisms, whilst implementation practicality necessitates cost reduction through technological iteration. Its primary benefit over other therapies is the capacity to develop a quantitative model of tactile-neural connection based on this approach; nonetheless, additional evidence-based medical research is required to standardize operating methods. Future research might focus on integrating with flexible electronic skin technologies to increase the accuracy of tactile information collecting.

### 4.5 The study's advantages and disadvantages

This study consolidated current literature to better understand the effectiveness features of manual therapy treatments as well as the contextual elements that influence their success. For the first time, it examined the features of existing manual therapy intervention modalities using the conceptual framework of manual medicine, with the goal of identifying similarities across multidimensional results and contributing factors. A manual medicine intervention strategy model was established, which, within the diagnostic and treatment system of “assessment-intervention-feedback,” constituted a biopsychosocial integrated therapy characterized by tactile mediation, dynamic regulation, and multi-system collaboration. This model served as a theoretical foundation for clinical practice and policy development, exhibiting gradual synergistic effects across four technical aspects throughout the course of the diagnostic and treatment cycle. However, the study has certain drawbacks.

First, the scope of the discovered effectiveness features and contextual elements may have been limited since it just contained literature from the patient's point of view and ignored the opinions of practitioners or other stakeholders. Second, grey literature was excluded despite the fact that eight databases were searched and typical manual treatment techniques were included; this might bring bias into the retrieval of information. Furthermore, the information gathered showed regional peculiarities, therefore localization must be taken into account when extrapolating results.

### 4.6 Implications for future research and practice

This study guides clinicians to leverage contextual factors to maximize the placebo effect in clinical practice, promotes patient-centered clinical implementation to improve clinical outcomes, and harnesses the advantages of holistic evaluation and individualized treatment. It improves patients' comprehension of the features of manual therapy methods, empowering them to choose the best course of action for their individual requirements. It supports focused healthcare delivery by giving decision-makers manual therapy diagnostic and treatment models. Future studies must take into account the features of manual therapy's intervention effects and work to thoroughly assess efficacy from biopsychosocial multi-dimensions in order to illustrate its therapeutic advantages. Future research should also examine the effects on efficacy of pertinent contextual elements, such as patient expectations and patient-practitioner interactions.

## 5 Conclusion

This study demonstrates that manual therapy enhances health outcomes via biopsychosocial pathways, and that patient-centered therapeutic partnerships and environmental design are critical to its effectiveness. In the future, it will be important to break down the existing “technology-centric” research paradigm and build an interdisciplinary study framework that incorporates patient experiences, neurobiological causes, and societal influences. Utilizing wearable technology (e.g., real-time physiological feedback monitoring) and artificial intelligence (e.g., effectiveness prediction models based on patient attributes) may open up new avenues for manual treatment optimization as precision medicine advances. There is a call for global health systems to re-evaluate the positioning of manual therapy, upgrading it from “complementary and alternative medicine” to a core component of chronic disease management and mental health promotion.

## Data Availability

The original contributions presented in the study are included in the article/Supplementary Material, further inquiries can be directed to the corresponding authors.
