# Supplementary material for: Patients’ experiences and perceived outcomes of manual therapy: a qualitative systematic review with framework synthesis
Source: Front Pain Res (Lausanne). 2026 Sep 11;7:1767395. doi: 10.3389/fpain.2026.1767395 (PMC13612508; doi:10.3389/fpain.2026.1767395)
Supplement: Supplementary file 1 [file Supplementaryfile1.pdf]

Appendices 1

Database	Search Strategy
China National Knowledge Infrastructure (CNKI)<br>743	TKA=('针灸'+'针刺'+'电针'+'体针'+'耳针+'头针''+'灸'+'按摩'+'推拿'+'点穴'+'穴位按压'+'拔罐'+'刮痧'+'埋线'+'穴位注射')*('定性研究'+'质性研究'+'访谈'+'民族志'+'人种学'+'扎根理论'+'现象学'+'行动研究法'+'混合研究') OR SU=('针灸'+'针刺'+'电针'+'体针'+'耳针+'头针''+'灸'+'按摩'+'推拿'+'点穴'+'穴位按压'+'拔罐'+'刮痧'+'埋线'+'穴位注射')*('定性研究'+'质性研究'+'访谈'+'民族志'+'人种学'+'扎根理论'+'现象学'+'行动研究法'+'混合研究')
Wanfang Data Knowledge Service Platform<br>685	题名或关键词:((″针灸″ or ″针刺″ or″电针″ or″体针″ or″耳针″ or″头针″ or ″灸″ or ″按摩″ or ″推拿″or ″点穴″or ″穴位按压″or″拔罐″or″刮痧″or″埋线″or″穴位注射″) and (″定性研究″ or ″质性研究″ or ″访谈″ or ″民族志″ or ″人种学″ or ″扎根理论″ or ″现象学″ or ″行动研究法″or ″混合研究″)) or 主题:((″针灸″ or ″针刺″ or″电针″ or″体针″ or″耳针″ or″头针″ or ″灸″ or ″按摩″ or ″推拿″or ″点穴″or ″穴位按压″or″拔罐″or″刮痧″or″埋线″or″穴位注射″) and (″定性研究″ or ″质性研究″ or ″访谈″ or ″民族志″ or ″人种学″ or ″扎根理论″ or ″现象学″ or ″行动研究法″or ″混合研究″))
VIP Chinese Journal Full-Text Database<br>630	(M=(针灸 OR 针刺 OR 电针 OR 体针 OR 耳针 OR 头针 OR 灸 OR 按摩 OR 推拿 OR 点穴 OR 穴位按压 OR 拔罐 OR 刮痧 OR 埋线 OR 穴位注射) AND (定性研究 OR 质性研究 OR 访谈 OR 民族志 OR 人种学 OR 扎根理论 OR 现象学 OR 行动研究法 OR 混合研究)) OR (R=(针灸 OR 针刺 OR 电针 OR 体针 OR 耳针 OR 头针 OR 灸 OR 按摩 OR 推拿 OR 点穴 OR 穴位按压 OR 拔罐 OR 刮痧 OR 埋线 OR 穴位注射) AND (定性研究 OR 质性研究 OR 访谈 OR 民族志 OR 人种学 OR 扎根理论 OR 现象学 OR 行动研究法 OR 混合研究))
China Biology Medicine (CBM) disc<br>289	(″针灸″[全部字段] OR (″针刺″[全部字段] OR ″药物针刺″[全部字段] OR ″针刺″[主题词]) OR (″电针″[全部字段] OR ″电针″[主题词]) OR ″体针″[全部字段] OR (″耳针″[全部字段] OR ″耳针刺″[全部字段] OR ″耳廓针刺术″[全部字段] OR ″耳廓针刺″[全部字段] OR ″针刺, 耳″[主题词]) OR (″头针″[全部字段] OR ″头针疗法″[全部字段] OR ″头皮针″[全部字段] OR ″头针疗法″[主题词]) OR ″灸″[全部字段] OR (″按摩″[全部字段] OR ″体区疗法″[全部字段] OR ″按摩″[主题词]) OR (″推拿″[全部字段] OR ″推拿疗法″[全部字段] OR ″推拿疗法″[主题词]) OR ″点穴″[全部字段] OR (″穴位按压″[全部字段] OR ″指压(Shiatsu)″[全部字段] OR ″指压(Zhi Ya)″[全部字段] OR ″指压(Chih Ya)″[全部字段] OR ″指压(Shiatzu)″[全部字段] OR ″穴位按压″[主题词]) OR (″拔罐″[全部字段] OR ″拔罐疗法″[全部字段] OR ″拔罐治疗″[全部字段] OR ″拔罐疗法″[主题词]) OR ″刮痧″[全部字段] OR (″埋线″[全部字段] OR ″穴位埋线疗法″[全部字段] OR ″穴位埋线疗法″[主题词]) OR (″穴位注射″[全部字段] OR ″水针疗法″[全部字段] OR ″水针″[全部字段] OR ″穴位注射疗法″[全部字段] OR ″水针疗法″[主题词])<br>) AND (″定性研究″[全部字段] OR ″质性研究″[全部字段] OR ″访谈″[全部字段] OR ″民族志″[全部字段] OR (″人种学″[全部字段] OR ″原始社

	会"[全部字段] OR "人种学"[主题词]) OR ("扎根理论"[全部字段] OR "扎根理论"[主题词]) OR "现象学"[全部字段] OR "行动研究法"[全部字段] OR "混合研究"[全部字段])
Web of Science<br>2404	#1 TS=(acupuncture OR manual acupuncture OR electroacupuncture OR body needling OR ear acupuncture OR auricular needling OR scalp acupuncture OR laser acupuncture OR moxibustion OR moxabustion OR moxa OR manipulation OR massage OR manual therapy OR acupressure OR reflexotherapy OR ayurvedic OR aromatherapy OR chiropractic OR osteopathic OR osteopath OR cranio - sacral therapy OR healing touch OR therapeutic touch OR myofascial release OR neuromuscular therapy OR shiatsu OR trigger point OR tuina OR chuna OR cupping OR scrapping therapy OR embedding thread OR acupoint embedding therapy OR acupoint injection)<br>#2 TS=("qualitative research" or "qualitative study" or "qualitative methodology" or "phenomenological research" or "grounded theory approach" or "ethnographic research" or "ethnographic study" or "historic research" or "historic study" or "action research" or "case study" or "mixed study" or "mixed research")<br>#3 TS=animal<br>#1 AND #2 NOT #3
PubMed<br>139	#1 "Acupuncture"[MeSH Terms] OR "Acupuncture Therapy"[MeSH Terms] OR "acupuncture, ear"[MeSH Terms] OR "Acupuncture Points"[MeSH Terms] OR "Acupuncture Analgesia"[MeSH Terms] OR "Electroacupuncture"[MeSH Terms] OR "Transcutaneous Electric Nerve Stimulation"[MeSH Terms] OR "Acupuncture"[Title/Abstract] OR "Electroacupuncture"[Title/Abstract] OR "body needling"[Title/Abstract] OR "Acupuncture Therapy"[Title/Abstract] OR "ear acupuncture"[Title/Abstract] OR "auricular needling"[Title/Abstract] OR "scalp acupuncture"[Title/Abstract] OR "laser acupuncture"[Title/Abstract] OR "transcutaneous electrical nerve stimulation"[Title/Abstract] OR "moxibustion"[MeSH Terms] OR "moxibustion"[Title/Abstract] OR "moxa"[Title/Abstract] OR "musculoskeletal manipulations"[MeSH Terms] OR "massage"[MeSH Terms] OR "acupressure"[MeSH Terms] OR "reflexotherapy"[MeSH Terms] OR "medicine, ayurvedic"[MeSH Terms] OR "manipulation, chiropractic"[MeSH Terms] OR "aromatherapy"[MeSH Terms] OR "manipulations musculoskeletal"[Title/Abstract] OR "Manipulation Therapy"[Title/Abstract] OR "Manipulative Therapies"[Title/Abstract] OR "Manipulative Therapy"[Title/Abstract] OR "therapies manipulative"[Title/Abstract] OR "therapy

Cochrane	manipulative"[Title/Abstract] OR "therapy manipulation"[Title/Abstract] OR "Manipulation Therapies"[Title/Abstract] OR "therapies manipulation"[Title/Abstract] OR "Reflexology"[Title/Abstract] OR "Rolfing"[Title/Abstract] OR "Manual Therapies"[Title/Abstract] OR "Manual Therapy"[Title/Abstract] OR "therapies manual"[Title/Abstract] OR "therapy manual"[Title/Abstract] OR "Zone Therapy"[Title/Abstract] OR "Zone Therapies"[Title/Abstract] OR "therapy zone"[Title/Abstract] OR "Massage Therapy"[Title/Abstract] OR "Massage Therapies"[Title/Abstract] OR "therapies massage"[Title/Abstract] OR "therapy massage"[Title/Abstract] OR "Shiatsu"[Title/Abstract] OR "Zhi Ya"[Title/Abstract] OR "Reflex Therapy"[Title/Abstract] OR "therapy reflex"[Title/Abstract] OR "Ayurvedic Medicine"[Title/Abstract] OR "Hindu Medicine"[Title/Abstract] OR "medicine hindu"[Title/Abstract] OR "Siddha Medicine"[Title/Abstract] OR "medicine siddha"[Title/Abstract] OR "Chiropractic Manipulation"[Title/Abstract] OR "spinal adjustment chiropractic"[Title/Abstract] OR "Chiropractic Spinal Adjustment"[Title/Abstract] OR "Chiropractic Spinal Adjustments"[Title/Abstract] OR "Chiropractic Adjustment"[Title/Abstract] OR "adjustment chiropractic"[Title/Abstract] OR "Aromatherapies"[Title/Abstract] OR "Aroma Therapy"[Title/Abstract] OR "Aroma Therapies"[Title/Abstract] OR "Tuina"[Title/Abstract] OR "chuna"[Title/Abstract] OR "cupping therapy"[MeSH Terms] OR "cupping"[Title/Abstract] OR "scrapping therapy"[Title/Abstract] OR "embedding thread"[Title/Abstract] OR "acupoint embedding therapy"[Title/Abstract] OR "acupoint injection"[Title/Abstract]<br>#2 ("mixed study"[Title/Abstract] OR "mixed research"[Title/Abstract] OR "qualitative research"[Title/Abstract] OR "qualitative study"[Title/Abstract] OR "qualitative methodology"[Title/Abstract] OR "phenomenological research"[Title/Abstract] OR "grounded theory approach"[Title/Abstract] OR "ethnographic research"[Title/Abstract] OR "ethnographic study"[Title/Abstract] OR "historic research"[Title/Abstract] OR "historic study"[Title/Abstract] OR "action research"[Title/Abstract] OR "case study"[Title/Abstract]) NOT "animals"[MeSH Terms]<br>#1 AND #2
Cochrane	#1 MeSH descriptor: [Acupuncture] explode all trees

library<br>2196	#2 MeSH descriptor: [Acupuncture Therapy] explode all trees<br>#3 ("acupuncture" OR "electroacupuncture" OR "body needling" OR "acupuncture therapy" OR "ear acupuncture" OR "auricular needling"):ti,ab,kw<br>#4 MeSH descriptor: [Moxibustion] explode all trees<br>#5 ("moxibustion" OR "moxa"):ti,ab,kw<br>#6 MeSH descriptor: [Massage] explode all trees<br>#7 MeSH descriptor: [Musculoskeletal Manipulations] explode all trees<br>#8 MeSH descriptor: [Acupressure] explode all trees<br>#9 MeSH descriptor: [Reflexotherapy] explode all trees<br>#10 MeSH descriptor: [Medicine, Ayurvedic] explode all trees<br>#11 MeSH descriptor: [Manipulation, Chiropractic] explode all trees<br>#12 MeSH descriptor: [Aromatherapy] explode all trees<br>#13 ("manipulation" OR "massage" OR "manual therapy" OR "acupressure" OR "reflexotherapy" OR "ayurvedic" OR "aromatherapy" OR "chiropractic" OR "osteopathic" OR "osteopath" OR "cranio-sacral therapy" OR "healing touch" OR "therapeutic touch" OR "myofascial release" OR "neuromuscular therapy" OR "shiatsu" OR "trigger point" OR "tuina" OR "chuna" OR "cupping" OR "scrapping therapy" OR "embedding thread" OR "acupoint embedding therapy" OR "acupoint injection"):ti,ab,kw<br>#14 #1 OR #2 OR #3 OR #4 OR #5 OR #6 OR #7 OR #8 OR #9 OR #10 OR #11 OR #12 OR #13<br>#15 ("qualitative research" OR "qualitative study" OR "qualitative methodology" OR "phenomenological research" OR "grounded theory approach" OR "ethnographic research" OR "ethnographic study" OR "historic research" OR "historic study" OR "action research" OR "case study" OR "mixed study" OR "mixed research"):ti,ab,kw<br>#16 MeSH descriptor: [Animals] explode all trees<br>#17 (animal):ti,ab,kw<br>#18 #16 OR #17<br>#19 #14 AND #15 NOT #18
Embase<br>136	#1 'acupuncture':ti,ab,kw OR 'electroacupuncture':ti,ab,kw OR 'body needling':ti,ab,kw OR 'acupuncture therapy':ti,ab,kw OR 'ear acupuncture':ti,ab,kw OR 'auricular needling':ti,ab,kw OR 'moxibustion':ti,ab,kw OR 'moxa':ti,ab,kw OR 'manipulation':ti,ab,kw OR 'massage':ti,ab,kw OR 'manual therapy':ti,ab,kw OR 'acupressure':ti,ab,kw OR 'reflexotherapy':ti,ab,kw OR 'ayurvedic':ti,ab,kw OR 'aromatherapy':ti,ab,kw OR 'chiropractic':ti,ab,kw

	OR 'osteopathic':ti,ab,kw OR 'osteopath':ti,ab,kw OR 'cranio - sacral therapy':ti,ab,kw OR 'healing touch':ti,ab,kw OR 'therapeutic touch':ti,ab,kw OR 'myofascial release':ti,ab,kw OR 'neuromuscular therapy':ti,ab,kw OR 'shiatsu':ti,ab,kw OR 'trigger point':ti,ab,kw OR 'tuina':ti,ab,kw OR 'chuna':ti,ab,kw OR 'cupping':ti,ab,kw OR 'scrapping therapy':ti,ab,kw OR 'embedding thread':ti,ab,kw OR 'acupoint embedding therapy':ti,ab,kw OR 'acupoint injection':ti,ab,kw<br>#2 ('qualitative research':ti,ab,kw OR 'qualitative study':ti,ab,kw OR 'qualitative methodology':ti,ab,kw OR 'phenomenological research':ti,ab,kw OR 'grounded theory approach':ti,ab,kw OR 'ethnographic research':ti,ab,kw OR 'ethnographic study':ti,ab,kw OR 'historic research':ti,ab,kw OR 'historic study':ti,ab,kw OR 'action research':ti,ab,kw OR 'case study':ti,ab,kw OR 'mixed study':ti,ab,kw OR 'mixed research':ti,ab,kw) NOT 'animal'/exp<br>#1 AND #2
